# From Human Needs to Value-Driven Preferences: Consumers’ Willingness to Participate in an Innovative Food Supply Chain Model

**DOI:** 10.3390/foods15020346

**Published:** 2026-01-17

**Authors:** Biancamaria Torquati, Chiara Paffarini, Giacomo Giulietti, Lucio Cecchini, Daniel Vecchiato, Francesco Musotti, Giordano Stella

**Affiliations:** 1 Department of Agricultural, Food and Environmental Sciences, University of Perugia, Borgo XX Giugno, 74, 06121 Perugia, Italy; bianca.torquati@unipg.it (B.T.); giacomo.giulietti@dottorandi.unipg.it (G.G.); luciocecchini89@gmail.com (L.C.); stellagiordano@libero.it (G.S.); 2 Department of Land, Environment, Agriculture and Forestry, University of Padova, Viale dell’ Università 16, 35020 Legnaro, Italy; daniel.vecchiato@unipd.it

**Keywords:** AFNs, human values, discrete choice modelling, Schwarz values, civil economy, sustainable food systems

## Abstract

Reflection on sustainable economic models, such as the civil economy, has led to the development of alternative food supply chains grounded in ethical values and practices. From this perspective, the Food Village model was proposed to meet stakeholders’ needs, overcome the limitations of Alternative Food Networks, and scale up. In this study, a Discrete Choice Experiment on hypothetical Food Village participation scenarios was combined with the Portrait Values Questionnaire to analyse preferences for the model’s attributes in relation to personal values. The results indicate that consumers appreciate the ethical and territorial characteristics of Food Village, such as local and organic products and cooperative governance, as long as convenience is guaranteed (product variety, flexible hours). Furthermore, they prefer moderate forms of participation, while excessively burdensome involvement reduces their willingness to participate. Individual values influence preferences: values of “self-transcendence” and conservation are associated with greater willingness, while those of “self-affirmation” correlate with lower adherence to Food Village. This evidence suggests implications for policy and scalability: initiatives like Food Village, if supported by public incentives and flexible participatory schemes, can contribute to more sustainable food systems at scale.

## 1. Introduction

Following the major environmental disasters that occurred during the last decades of the twentieth century and the simultaneous emergence of ecological movements, the debate on the sustainability of the prevailing economic growth model, until then considered the sole paradigm of reference, began to take shape. This debate gained political recognition in 1987, when the concept of sustainable development was formally introduced in the report Our Common Future by the World Commission on Environment and Development [1].

Subsequently, the discussion on the need to transform the dominant economic paradigm stimulated several fields of reflection, paving the way for the development of heterodox economic models [2,3]. These models have since served as conceptual and operational frameworks to inspire and support processes of change through the implementation of sustainable practices. Among these models, we highlight the Bioeconomy [4], Fair Trade Economy [5,6], Ecological Economics [7], Civil Economy (CE) [8], the Economy of Happiness [9], the Human Scale Development (H-SD) theory [10], and, more recently, the Economy for the Common Good (ECG) [11,12].

This reflection process has also influenced the way food chains are conceived. In fact, with the aim of overcoming the traditional growth-oriented model applied to the food system [13], new forms of food supply and distribution, known as Alternative Food Networks (AFNs), have emerged over the last three decades.

Among the many definitions proposed in the literature, AFNs can be described “as emerging networks of producers, consumers, and other actors that are alternatives to the more standardized industrial mode of food supply” [14].

During this period, several organizational forms have developed and become recognized as AFNs, including Community Supported Agriculture (CSA), Farmers’ Markets, direct sales, community gardening initiatives [15], Italian Solidarity Purchase Groups (SPGs), Organized Groups of Supply and Demand (OGSD) [16], and the Food Coop Park Slope (FCPS) model.

Although these initiatives have promoted an eco-sustainable paradigm shift in the food chain, AFNs have largely remained niche phenomena [17], due to several structural limitations. The most significant include the inability to fully meet consumer demand in terms of product variety, the requirement for a medium-to-high level of consumer involvement, limited accessibility in terms of purchasing hours, and often uncompetitive prices.

According to Stella et al. [18], to emerge from the niche and achieve a “jump in scale” [19], AFNs must be able to respond to the needs of all stakeholders involved along the supply chain. Building on these considerations, an innovative AFN model called “Food Village” (FV) has been proposed [18]. The Food Village (FV) represents an innovative model of the food supply chain, developed based on Max-Neef’s Needs Matrix [20], with the aim of satisfying the needs of all individuals involved in and affected by the food system.

The design of such an innovative model necessarily required an investigation into the extent to which the distinctive features of the FV could be valued and appreciated by consumers, and how these perceptions relate to their underlying psychological attitudes. Accordingly, this study addresses two main research questions: (1) to assess consumer attitudes toward the constitutive attributes of the FV model; and (2) to examine the role of individual human values in shaping consumer preferences for the FV model.

To address the first research question and considering that the design of a new program or product necessarily requires an understanding of public preferences, we applied the Discrete Choice Experiment (DCE) methodology. This approach has been widely used to investigate preferences for programs or products that are not yet observable in the market (among others, [21,22,23]).

Human values were measured using the Schwartz Portrait Values Questionnaire (PVQ) [24]. According to Schwartz’s theory of basic human values [24], values are enduring beliefs—emotionally grounded and abstract—that transcend specific actions and situations. The PVQ was developed by Schwartz to operationalize individual value orientations through four higher-order dimensions: self-enhancement, self-transcendence, openness to change, and conservation. This instrument enables the connection between stable individual value systems and context-specific attitudes or preferences.

Despite the growing body of literature on AFNs, few studies have explored the psychological and value-based determinants that shape consumer preferences for innovative food system models. Most existing research has focused on the socio-economic and organizational dimensions of AFNs, emphasizing aspects such as governance structures, short supply chains, or environmental performance [25,26]. However, a limited number of empirical studies have examined how individual human values influence consumer acceptance of new forms of collective and sustainable food organization.

This study contributes to filling this gap by integrating the DCE methodology with Schwartz’s theory of basic human values, thereby linking behavioural and ethical dimensions of food consumption with structural innovations in AFNs. Through the case of the FV, the research provides novel insights into how consumers evaluate alternative configurations of the food supply chain and how their value orientations (e.g., self-transcendence or conservation) drive these preferences.

By doing so, this paper offers a twofold contribution. First, it expands the analytical framework of AFNs by incorporating a human-values perspective, thus connecting individual-level psychology with systemic food innovation. Second, it provides policy-relevant evidence for designing participatory and value-oriented food networks capable of scaling up from niche experiences to broader models of sustainable local development.

The paper is organized in the following manner. In Section 1.1 subparagraph, we present the concepts of needs, values, and motivations behind food consumption and their measurement, and the relevant literature on the topic, while in Section 1.2. we introduced the new proposal of Food Village within the framework of AFNs. In Section 2 “Materials and Methods”, firstly in Section 2.1, we specifically describe the Food Village model case study, while in Section 2.2, we explain the phases of the research (in terms of Definition of attributes and levels of provision, Experimental Design used, Questionnaire Development, Data collection, and Random parameters Logit—RPL model used). In Section 3 “Results,” we present the value reliability (in the Section 3.1 subparagraph), the Principal Component Analysis (PCA) (in the Section 3.2 subparagraph), and the RPL Model results (in Section 3.3 subparagraph). Section 4 shows the discussion, while Section 5 shows the conclusion.

### 1.1. Needs, Values and Motivations Behind Food Consumption and Their Measurement

Over the past decades, the evolution of food consumption patterns has been accompanied by increasing scholarly attention to the psychological and social factors that influence consumer choices.

Several studies have revealed a strong relationship between human values, social dynamics, and purchasing behaviour [27].

Some authors suggest that introducing more stable elements, such as personal values, into consumer analyses could be one possible way to make preference studies more realistic and reliable [27,28]. Several scholars have recognized values as crucial predictors of food consumption behaviour [29,30,31,32,33,34]. Nevertheless, only a limited number of studies have explicitly examined the influence of personal human values on consumers’ purchasing decisions [35].

In the last fifty years, the social sciences applied to economics have increasingly emphasized the importance of values, particularly in explaining consumer behaviour [36]. Various theoretical approaches and analytical tools have been developed to study this relationship [37,38,39,40]. Among the different conceptual frameworks and measurement instruments designed to identify how individual values and beliefs influence food choices, Schwartz’s Theory of Basic Human Values [41] is widely recognized as one of the most structured and empirically validated. Compared with earlier models such as the Rokeach Value Survey [39] and the Social Values Inventory [42], Schwartz’s framework provides a more comprehensive and consistent theoretical foundation, as underlined by [43]. Furthermore, several authors have adopted Schwartz’s theory in empirical studies on consumer food behaviour, confirming its reliability and explanatory power [28,35,44,45].

Schwartz’s Theory of Basic Human Values provides a complementary perspective to Max-Neef’s Human-Scale Development (H-SD) framework [10], which, starting in the 1980s, contributed to re-conceptualizing the economic development process in terms of human well-being [46]. The Economics of Human-Scale Development argues that the economic system should respond to human needs, which are classified into two categories: existential (“being”, “having”, “doing”, and “interacting”) and axiological (subsistence, protection, affection, understanding, participation, creativity, identity, and freedom) [20,47]. While Max-Neef identifies universal human needs, Schwartz’s model helps to explain how and why individuals prioritize and pursue these needs based on their underlying value orientations.

From this perspective, personal food values, shaped by both individual and social factors, strongly influence food choice behaviour [48,49]. Beyond the satisfaction of material needs, food purchasing act thus becomes a means through which individuals express their identity, cultural background, and sense of belonging to a specific social context [50,51,52].

According to the motivation that underlies each value Schwartz’s theory defines 10 basic values that guide human behaviour and could be differentiated into two pairs of opposing categories: self-enhancement (hedonism, achievement and power) in opposition to self-transcendence (benevolence and universalism); conservation (security, conformity and tradition) versus openness to change (stimulation, self-direction) [28,53].

Schwartz [54] (p. 21) defined value as “a desirable trans-situational goal varying in importance, which serves as a guiding principle in the life of a person or other social entity”. The values of Schwartz’s theory are grounded on three universal requirements of human existence that “are the needs of individuals as biological organisms, requisites of coordinated social interaction, and the survival and welfare needs of groups” [53] (p. 4). Based on the research objective and the sample being studied, two different approaches to measuring individual values were suggested by Schwartz: the Schwartz Value Survey (SVS) and the PVQ [24].

The PVQ approach is recognized as being easier to apply and more dependable [53], and it is employed in the present study. Compared to the original version, which presented 40 items (affirmations), the model adopted for our study contains 21 items [55,56].

The PVQ, the one with 21 items, aims to reduce cognitive complexity by presenting interviewees with brief verbal portraits of different people, including their goals, aspirations, or desires, which implicitly indicate the importance of a single value [57].

This short version has demonstrated acceptable equivalence of meaning across cultures and considerable predictive validity [58,59]. The PVQ is a relatively recent way. According to Schwartz, the PVQ aims to reduce cognitive complexity by presenting interviewees with brief verbal portraits of different people, which implicitly convey the person’s goals, aspirations, or desires, thereby indicating the importance of a single value [60] (see Appendix A for the full Schwartz values and defining goals, Table A1).

Scholars employing Schwartz’s theory of basic human values have also used Discrete Choice Experiments (DCEs) to investigate how personal values influence consumer purchasing decisions.

Next to the personal values and characteristics, the consumer’s choice behaviour is obviously related to the product or service characteristics that respond to human needs in different degrees, according to their specifics and attributes [47].

These characteristics can be considered condensations of what drives consumption. It, therefore, becomes essential to measure how much the attributes of a product or service motivate the consumer’s choice. At this purpose, DCEs are used in this study. Indeed, among the methods of analysis of “stated preferences”, the use of DCEs is amply justified according to the literature, in which this approach has been the most adopted for the evaluation of the influence of “credence” attributes [61,62,63].

Caracciolo et al. (2016) [28] adopted this perspective to assess the relationship between Schwartz’s human values and consumer preferences in five European countries regarding process attributes of pig farming, using a generalized logit model. In addition to highlighting consumers’ high awareness of the environmental impact of pig production, the authors emphasized that individual values strongly influence purchasing choices.

Using non-hypothetical experimental auctions, Pomarici et al. [64] demonstrated that seven out of eight of Schwartz’s value dimensions were statistically significant in explaining wine consumers’ willingness to pay (WTP) for the three wines considered in their study.

Contini et al. [65] analysed how the use of local-origin products could influence restaurant consumers in Italy and Germany through a choice experiment framed within the Schwartz value system. In this study, the Schwartz framework proved useful for defining consumer clusters, particularly the so-called “locavores”, characterized by a strong preference for local food. This cluster was distinguished by high levels of conservation, self-enhancement, and stimulation.

Fitzsimmons and Cicia [35] focused on the role of human values in influencing consumers’ willingness to pay for the social outcomes of extrinsic credence attributes when purchasing early potatoes in Italy and Germany. Their findings confirmed a relationship between consumers’ identification with cross-cultural human values, as defined in Schwartz’s framework, and their willingness to pay for specific product attributes. Overall, the literature confirms both the relationship between food habits and individuals’ systems of human values and the need to further deepen research on this topic.

The theoretical link between human needs and values provides the conceptual foundation for the present analysis. In this study, the FV model is used as a case study to explore how individual value orientations and related motivations influence consumer preferences towards an innovative form of AFN.

### 1.2. Alternative Food Networks and the New Proposal of Food Village

The alternative food practices named AFNs emerged in the 1990s as a reaction against the agro-industrial system impacts in terms of both environmental such as biodiversity reduction, land degradation, greenhouse gas emissions, and air pollution [66], and social as the reduction in income, increased phenomena of farm failure, farmer marginalisation, and land abandonment [67].

AFNs represent a “model of resistance” to the disembedded, corporate-dominated agrifood regime, seeking to re-embed production and consumption at the local level, re-socialize relationships in the food chain, and re-localize food economies [68].

These initiatives aim to promote more sustainable practices (both environmentally and socially) while ensuring fair economic returns for producers. Over the past three decades, a rich body of literature has explored the evolution and values of AFNs. No single, comprehensive definition is universally accepted, since “alternative food network” serves as an umbrella term for diverse practices that differentiate themselves from the conventional, industrial model [68].

One of the earliest definitions by Feenstra [69] described AFNs as food systems “rooted in particular places” that aim to be economically viable for farmers and consumers, use ecologically sound production and distribution, and enhance social equity and democracy in communities. Subsequent scholars similarly emphasize how AFNs reconnect producers and consumers through shortened supply chains, fostering trust and transparency in contrast to anonymous global markets [70].

In general, AFNs include any form of food production, distribution, or retail that operates “far from the dominant (or conventional) logic of the market” [71] (p. 1). A key feature is the minimization of intermediaries: AFNs typically involve short food supply chains, meaning fewer steps between farmer and consumer, which helps redistribute value to producers and build closer social connections [68]. Studies note that these networks seek to provide local, high-quality, or organic food at a fair price, often invoking ethical commitments like animal welfare, environmental stewardship, and community empowerment [70].

AFNs have proliferated in many forms, leading to increasing differentiation among initiatives [68].

Examples of AFN models around the world include farmers’ markets (direct producer-to-consumer sales), CSA schemes, food cooperatives and solidarity purchasing groups, local organic and agroecological farming networks, fair trade initiatives, and urban community gardens, among others [72].

AFN consumers are typically characterized by higher education levels, a strong orientation toward food quality and health, and motivations rooted in trust, social interaction, and support for local producers, rather than price sensitivity [73,74]. Across European contexts, these consumers are often middle-aged or older, more frequently female, and display shared ethical and civic values, viewing AFN participation as both an individual consumption choice and a form of collective action aligned with sustainability and local development goals [74,75].

Recent empirical studies indicate that consumers who participate in AFNs are strongly driven by sustainability-related goals encompassing both environmental concerns and ethical values [74,76,77]. These motivations manifest in pro-sustainability consumer behaviours, as AFN participants commonly seek to minimize their ecological footprint through local and organic food purchases while also supporting fair trade, animal welfare, and small-scale producers as part of their ethical commitment [78]. Across all contexts, AFNs share a common ethos: they seek to “re-spatialize and re-socialize food production, distribution and consumption” [15], rebuilding food systems that are more environmentally sustainable and socially just. AFNs hold potential to improve farmers’ livelihoods and empower consumers, though debates continue about the magnitude of their impact and how to scale up these alternatives without losing their core values [70].

As other authors previously questioned [17,25], Stella et al. [18] recently investigated the extent to which these models are truly alternative. These authors pointed out that, until now, AFNs have failed to achieve a scale change and therefore have not become a real alternative to the large-scale retail trade.

Starting from these considerations, Stella et al. [18] hypothesize that to make the AFNs make a change of scale, it is necessary to respond simultaneously to all the needs of the stakeholders involved. To devise a new model of AFN capable of achieving this goal, Stella et al. [18] used the needs matrix of Max-Neef et al. [47], (p. 33); the proposal of the FV emerged from this analysis.

The FV is an innovative food chain model based on the construction of a Food Community where the needs of all interested and involved people can be satisfied.

## 2. Materials and Methods

### 2.1. Identification and Description of the Case Study: The Food Village Model

The FV represents an innovative model of an AFN aimed at fostering food resilience, health, environmental care, social aggregation, and cultural biodiversity. Its goal is to create a “Food Community” in which the needs of all stakeholders—consumers, producers, and workers—are satisfied through a fair, ecological, and participatory agri-food system. The model integrates principles from the Ecological, Civil, Common Good, and Happiness economies, promoting efficiency, redistribution, and relationality in the pursuit of the common good. For a more detailed discussion of the economic models underpinning this new food supply chain model, see Stella et al. [18].

At the core of the FV model lies the “Community Pact for Food”, a set of shared values and practices that guide production, consumption, and environmental impact. The model is legally structured as a “Community Cooperative”, bringing together local producers, consumers, and employees within the same economic entity that manages production, processing, and distribution. The cooperative’s Food Market serves as the operational hub.

To support small and micro local farms, preference will be given to organic and locally produced products, and modular micro-processing plants (e.g., mills, dairies, oil mills, fruit and vegetable transformation units) are provided to increase added value and reduce logistical costs. Participatory democracy spaces within the FV promote co-planning of production and prices, offer consumer members the opportunity to work within the cooperative, promote participatory certification, and educational activities for both producers and consumers. Consumer members can access discounts linked to their level of participation in cooperative activities.

The FV further acts as a solidarity community, promoting inclusion, interculturality, and initiatives addressing food poverty and human rights. It adheres to the ECG framework and prepares an annual Common Good Balance Sheet to assess and improve its social, environmental, and economic impact.

### 2.2. The Phases of the Research

Consumers’ preferences for the attributes of the FV model were analysed by explicitly estimating preference structures according to respondents’ Schwartz Human Value (SHV) profiles and by modelling the distribution of these preference profiles using a Random Parameters Logit (RPL) model. To provide a concise overview of the methodological pathway, Table 1 illustrates the main steps followed in this research.

The study was articulated into four consecutive phases.

The first phase involved the selection of the case study, represented by the FV model. The second phase focused on the DCE. It began with the definition of the key attributes and levels of provision, which served as the basis for the experiment, and continued with the experimental design, including: the preliminary orthogonal design, its testing, the estimation of priors, and the development of the final D-efficient design. This phase also included the development of the questionnaire, which integrated the PVQ to capture individual value orientations, and the definition of the sampling strategy. The third phase consisted of the questionnaire survey, which collected data on respondents’ choices, socio-demographic characteristics, and human value profiles. Finally, the fourth phase concerned data analysis, including the reliability testing of the PVQ, a Principal Component Analysis (PCA) with varimax rotation to identify value dimensions, and the RPL estimation to model consumer preferences and heterogeneity. Concerning this last phase, we first assessed the reliability of the PVQ results. Once satisfactory reliability was confirmed, a PCA with varimax rotation was performed to reduce the number of variables and identify the main human value dimensions. The resulting component weights were then used to construct individual value profiles for each respondent. These individual-level value scores were subsequently included, together with the FV attributes, in the RPL model to capture preference heterogeneity across different Schwartz value profiles.

This approach allowed us to integrate psychological value orientations into the econometric analysis, thereby linking individual motivations with preferences for the FV model.

#### 2.2.1. Definition of Attributes and Levels of Provision

The DCE approach [79] was used to investigate consumers’ preferences for the FV model. This method is the most used approach for the analysis of the agribusiness multi-attributes products [80] demand and evaluation of credence attributes’ influence, among the analysis methods of stated preferences [81,82,83].

The DCE methodology consists of requesting consumers to respond to a questionnaire choosing the product or service that they prefer among those presented in each choice set; this differs from each other by the levels of product or service attributes. The status quo can be introduced as an explicit option, defined on relevant attributes [84], or as a “no-choice” option [85]. If the status quo is described on attributes, it can emerge either as one of the possible alternatives or as a fixed alternative (benchmark) in each core of choice.

The analysis is structured on repeated choices made on choice kernels composed of at least two alternatives (choice sets), from which the data necessary for estimating utility measures and WTPs are obtained [80].

By observing how people change their preferred option in response to changes in attribute levels, it is possible to determine their preferences with respect to attributes. In this way, it is possible to understand what the value (utility) is attributed to each element. Levels ranges can be quantitative or qualitative; for example, the price attribute is quantitative, and its levels are the various prices while the production system is qualitative, and its levels can be “organic” or “conventional”. Analysis based on quantitative levels has advantages in terms of modelling and evaluation of attributes and should therefore be preferred [86]. Quantitative levels can be presented either in absolute terms or as a difference from the status quo.

In line with the objectives of the study, five attributes describing the FV model were selected for the DCE. Two attributes refer to the structural and organizational characteristics of the Food Market, namely the opening days per week and the product assortment. Two additional attributes concern the type of products (organic or non-organic) and their origin (local or non-local). The fifth attribute relates to the level of consumer-member involvement in the cooperative’s activities (Table 2).

Each attribute was defined according to the main structural and functional characteristics of the FV model (see Stella et al. [18]).

Regarding the first attribute, we considered the number of opening days per week, such as 1, 3, 5, or 7 days per week.

As regards the second attribute, we considered three levels, starting from the design classification of food distribution chains [87]. The level of assortment is directly related to the square meters of product display. In other words, as the square meters increase, the linear meters dedicated to each commercial category increase, and consequently, the product variability offered for each. Therefore, we considered: a high level, corresponding to the assortment typically found in a hypermarket; medium level, corresponding to the assortment typically found in a supermarket; and low level, corresponding to the assortment typically found in a mini market. The third attribute concerns the share of certified organic products or products guaranteed through a Participatory Guarantee System (PGS). The PGS represents an alternative certification process directly managed by both producer and consumer members, ensuring transparency and trust in production practices. This attribute expresses the percentage of such certified or guaranteed products out of the total food products available in the Food Market, with three possible levels: less than 50%, more than 50%, and 100%. The fourth attribute refers to the proportion of locally sourced food products, defined as those produced within a maximum distance of 50 kilometres from the Food Market. This attribute reflects the degree of local embeddedness of the food supply chain and the emphasis placed on proximity between producers and consumers. Two levels were considered: less than 50% and more than 50% of the total food products available.

The fifth attribute, and arguably the most complex and central to the FV model, concerns the degree of consumer-member involvement in the organisation and management of the Food Market, as well as the related discount system. As already stated in the previous paragraph, the model provides that consumers can participate in two main ways: (i) by co-planning agricultural production together with farmers, and (ii) by contributing to in-store activities within the Food Market.

The co-planning of production involves consumers, once a year, in a guided process aimed at analysing their average food consumption and jointly deciding with farmers which crops to cultivate. Consumers participate by covering in advance 100% of the production costs of the raw materials (e.g., wheat for bread, olives for oil) and then regularly purchasing the co-planned products throughout the year. This form of participation is considered equivalent to one hour of work per month and is rewarded with a 10% discount on the corresponding expenditure, referred to as the “co-planned shopping”. While participation through in-store work requires consumer-members to dedicate three hours of work per month within the Food Market, contributing to sales-related activities such as product placement and basic maintenance tasks. This participation entitles them to a 20% discount on their regular purchases in the store that are not co-designed; this is referred to as “standard shopping”. Regarding this attribute, four levels of participation were therefore considered: none, no involvement and no discount; medium-low, participation in co-planning activities (equivalent to 1 h of work per month), advance payment of 100% of the production costs of the raw materials, and a 10% discount on co-planned shopping; medium-high, participation in in-store work (3 h per month) with a 20% discount on standard shopping; and high, participation in both activities (4 h of work per month in total), advance payment of 100% of the production costs for co-planned products, a 30% discount on co-planned shopping, and a 20% discount on standard shopping (Table 3).

#### 2.2.2. Experimental Design

The combination of the five attributes and their defined levels provided the basis for constructing the DCE. The experimental design aimed to elicit consumers’ preferences and to assess how different configurations of the FV model could influence their willingness to participate.

An unlabelled D-efficient design [88] was adopted for this analysis, generated using the *dcreate* module in STATA and based on the modified Fedorov algorithm [89,90,91]. The final design consisted of 24 choice sets, divided into four blocks of six choice sets each. Each respondent was therefore presented with six choice tasks, and in each task, asked to select the preferred alternative among three hypothetical FV models characterised by different combinations of the five attributes described above. In Table 4, an example of a choice task is reported.

In addition, a “no-choice” option was included. To improve the statistical efficiency of the design, a pilot survey was conducted with approximately 10% of the final sample to obtain preliminary estimates of the model coefficients (priors), which were subsequently used to generate the final design employed in the main survey [79].

#### 2.2.3. Questionnaire Development

The questionnaire used for data collection consisted of several sections, including:

(a) an introductory information box providing a detailed description of the FV model and the attributes and levels included in the DCE.
(b) the presentation of the six choice tasks used in the DCE.
(c) questions on respondents’ food purchasing habits and their perceptions of environmental and social issues.
(d) the 21-item Schwartz PVQ, used to measure individual human values [24]. (see Appendix A for the full PVQ, Table A2); and
(e) questions on respondents’ socio-demographic characteristics.

Considering the innovative character of the proposed model, section (a) consisted of an information box intended to provide respondents with a clear understanding of the FV model and of the conceptual foundations behind it. It introduced core ideas such as Community Cooperatives, Food Sovereignty, the CE, and the ECG. To mitigate the potential risks of cognitive overload and hypothetical bias associated with the complexity of these participatory mechanisms, respondents were required to spend a minimum of 12 min reading the informational materials before proceeding to the questionnaire. Moreover, participants were explicitly instructed to carefully read the descriptions of all attributes and their hypothetical levels before completing the choice tasks. These precautions, combined with respondents’ familiarity with online survey-based research, helped ensure a satisfactory level of comprehension and engagement with the experimental design. Consistently, only 9% of the completed questionnaires were excluded due to clearly incoherent or inconsistent response patterns.

Section (b) focused on the DCE presenting the choice sets, each characterised by different combinations of the five attributes described above. The aim was to quantify the trade-offs respondents made among attributes and levels, to identify the relative importance of each attribute in determining respondents’ choices and, consequently, their willingness to participate in an innovative food supply chain model.

Section (c) collected information on respondents’ food purchasing habits and perceptions of environmental and social issues. Questions covered the respondent’s role in household food purchases, membership in AFNs, shopping frequency and distance, average expenditure, and the share of organic food purchases. Respondents also rated the importance of various product attributes and reported their motivations or barriers to buying Fair-Trade products.

Section (d) of the questionnaire included the PVQ, consisting of 21 items, each presented as a brief description of a person. For example: “He/she strongly believes that people should care for nature. Looking after the environment is important to him/her.” Respondents were asked to indicate how similar the described person was to themselves using a six-point scale, where 1 means “very much like me” and 6 means “not like me at all”. The last section included socio-demographic characteristics of the respondents.

#### 2.2.4. Data Collection

A self-administered, structured electronic survey was developed to investigate consumer characteristics, including individual values and preferences, to assess how different configurations of the FV model could influence their willingness to participate. The survey was conducted in 2022 through an online access panel with a sample of 440 individuals recruited by a professional research agency (Demetra opinioni.net Srl, Venice, Italy). A non-probability quota sampling design was adopted, with quotas defined by age group and gender. Territorial representativeness was ensured by allocating respondents across five geographical macro-areas (North-West, North-East, Centre, South, and Islands).

After data cleaning, 38 respondents were excluded due to repetitive answers in several sections of the questionnaire, indicating) a poor understanding of the survey content. Therefore, the final sample consisted of 402 valid observations. Table 5 summarizes the main socio-demographic characteristics of the respondents.

A total of 60% of respondents reported being solely responsible for their household food purchases. Membership in AFNs is still limited: only 6.5% of respondents are members of SPGs, 4.2% participate in OGSDs, and 5% are members of CSA initiatives. Hypermarkets and supermarkets are the most used food retail channels, followed—at a much lower rate—by discount stores and traditional food shops. Among short supply chains, the local market is the most frequently used, although with significantly lower percentages compared to large-scale retail outlets. Regarding shopping distances, almost half of the respondents (approximately 50%) reported travelling between 1 and 5 km to purchase food; 21% travel shorter distances (less than 1 km), another 21% travel between 5 and 10 km, while only 8% travel more than 10 km. In terms of shopping frequency, 51% of respondents purchase food more than once a week, 33% once a week, and 8% every day. Smaller shares go shopping every two weeks (6%) or every three weeks (2%). Respondents reported spending an average of €394 per month on food for their households, of which approximately €26 is allocated to organic products. When asked about the factors influencing their food choices, respondents ranked sensory characteristics, health and hygiene safety, and special offers as the most important criteria. Trust in the producer, product origin (local, regional, national), and price were also considered relevant, albeit to a slightly lesser extent.

These results highlight a predominance of conventional purchasing channels, alongside a still limited but emerging interest in alternative and more sustainable food networks.

#### 2.2.5. Random Parameters Logit (RPL) Model

The data collected through the DCEs were analysed with a RPL [92,93], which considers the heterogeneity of consumer preferences by estimating specific and random individual parameters. The main advantages of such a model are that it allows for the consideration of heterogeneity in preferences not dependent on socio-economic aspects of individuals and is able to explain the correlation in the random variation of preferences and in unrestricted substitution models [93]. Based on the estimates obtained through RPL, it is possible to efficiently evaluate the behaviour of consumers and test to what extent some consumers might prefer certain attributes, while others do not.

According to the linear random utility framework proposed by Train (2009) [93], the utility of the respondent “*i*”, associated with each alternative “*j*”, within the set of choices “*n*” could be specified as follows:

(1) $$U_{ijn}=\beta_{i}\cdot x_{ijn}+\varepsilon_{ijn}$$

where “*x_ijn_*” represents the vector of attributes that characterize the alternative “*j*”, respectively for each participant “*i*” and choice set “*n*”; “***β***_*i*_” is a vector of parameters to be estimated which is assumed to be specific to the individual and constant among the alternatives; “*ε_ijn_*” represents the random error component that varies according to individuals, alternatives, and the choice set.

Data from DCEs questionnaire were analysed using the python package ‘xlogit’ [94] for RPL models. According to Train (2009) [93], the maximization of the simulated log-likelihood function procedure with 1000 Halton draws for the simulation was used to estimate the RPL coefficients, which are assumed to be independent and normally distributed.

It should be emphasized that the hypothetical biases (strategic behaviour) are a recurring problem in the design of stated preferences studies, including DCEs. Strategic behaviour can derive from the examination of choice scenarios to find the best one [95,96]. Ultimately, it is important to try to lighten as much as possible the cognitive load of the interviewees (with the right number of attributes and alternatives) and to consider any bias. The mitigation of hypothetical biases is crucial especially when stated preference methods are applied to derive welfare measures, namely, to estimate willingness to pay (WTP) or premium prices in market simulations. In fact, hypothetical biases might be more relevant when asking study participant their WTP in hypothetical markets. In our study this problem is mitigated by the fact that we did not include questions about purchase situations, and therefore the application of the DCE is in a context that by itself is less prone to hypothetical biases.

The inclusion of the SHV profiles in the RPL specification enabled the identification of systematic preference variations associated with individual human value orientations.

## 3. Results

### 3.1. Value Reliability

To assess the reliability of the PVQ results, Cronbach’s alpha coefficients were computed for each of the ten Schwartz values. For nine of these values, reliability was based on the responses to two PVQ items, while for Universalism it was calculated from three items [24,35,97].

The resulting alpha scores are reported in Table 6. According to the literature, alpha values above 0.50 are generally considered to indicate moderate to high reliability.

Acceptable levels of internal consistency (α > 0.70) were obtained for Achievement (0.76), Hedonism (0.78), and Universalism (0.71).

Power (0.62), Stimulation (0.59), Security (0.55), Benevolence (0.52), and Self-direction (0.52) showed moderate reliability, while Conformity (0.07) and Tradition (0.01) displayed low coefficients.

These lower values may reflect either the limited number of items per scale (two per value, except for Universalism) or cultural and contextual factors influencing how respondents interpret these specific value dimensions.

We retained the two values with low coefficients; however, their inclusion had a negligible impact on the subsequent analyses [35]. Despite their low alpha-values, their inclusion was necessary to maintain the theoretical coverage of the ‘Conservation’ meta-value of the PVQ. Excluding them would have impacted in the subsequent Principal Component Analysis, biasing the first principal component toward Security alone. The fact that they successfully loaded onto the first ‘Conservation’ component suggests that the underlying motivational goal was captured, and the subsequent model interactions rely on this more stable aggregate measure rather than the individual sub-scales.

Following the reliability assessment, a PCA was conducted to identify the underlying value dimensions among respondents.

### 3.2. Principal Component Analysis (PCA)

To reduce the number of variables and obtain component scores to be used as regressors in the RPL model, we conducted a PCA of the 21 PVQ items, using varimax rotation.

According to the SHV framework, the ten basic human values are expected to cluster into five higher-order meta-values. The first dimension, Openness to Change (stimulation, self-direction, and hedonism), reflects independence of thought and action, and a readiness for new experiences, as opposed to Conservation (security, conformity, and tradition), which emphasizes order, self-restriction, preservation of the past, and resistance to change. The second bipolar dimension contrasts Self-Transcendence (benevolence and universalism), oriented toward the welfare of others, with Self-Enhancement (achievement and power), focused on the pursuit of personal success, status, and dominance over others. Finally, Hedonism, while closely related to Openness to Change, can also stand as a distinct value, as it encompasses the individual search for pleasure and sensuous gratification for oneself [53,98]

The PCA with varimax rotation extracted three components with eigenvalues greater than one, jointly explaining 69.86% of the total variance.

The interpretation of the PCA components follows Schwartz’s theory of basic human values, according to which individual values cluster around higher-order motivational dimensions. Accordingly, each component was labelled based on the values showing the highest loadings and their shared motivational goals, rather than on individual items. Mixed loadings were interpreted as reflecting context-specific combinations of value orientations, consistent with previous empirical applications of the PVQ.

These components broadly align with the theoretical higher-order dimensions proposed in Schwartz’s framework, albeit with some context-specific nuances (Table 7).

The first component (Conservation/Self-transcendence) groups Security (0.808), Conformity (0.755), Benevolence (0.682), and Tradition (0.654), with a moderate contribution from Universalism (0.507). It represents values related to social order, stability, and the welfare of close others, emphasizing the preservation of established norms and collective well-being. This component reflects a segment of consumers who prioritize harmony, respect for rules, and relational trust.

The second component (Self-enhancement) shows strong loadings on Power (0.851), Achievement (0.817), and Hedonism (0.696). It captures values associated with status, personal success, and pleasure, indicating respondents who are motivated by self-realization, recognition, and competitive performance.

The third component (Openness to change/Self-transcendence) combines Self-direction (0.767), Stimulation (0.734), and Universalism (0.669). This component expresses an orientation toward autonomy, curiosity, and novelty, coupled with universalistic concerns such as tolerance, equality, and environmental protection. It thus represents consumers who are open to innovation, global awareness, and personal growth.

### 3.3. RPL Model Results

The data collected with the DCE were analysed by implementing a RPL model [93], which considers the heterogeneity of consumer preferences by estimating specific and random individual parameters. Table 8 reports the results obtained, estimated using the three value-based components identified through the PCA. The model converged successfully after 224 iterations, showing robust estimation statistics (Log-Likelihood = –2804.18; AIC = 5768.36; BIC = 6231.41).

The RPL results offer a clear overview of how respondents evaluate the key attributes of the FV model. Overall, the coefficients indicate that both practical and ethical dimensions significantly influence preferences. The following discussion details the contribution of each attribute to consumers’ utility and willingness to participate.

The coefficient for opening days per week is positive and highly significant (0.136; *p* < 0.001), suggesting that consumers clearly prefer configurations where the FV is open for more days per week. Accessibility and convenience in time management, therefore, play a crucial role in shaping consumer preferences.

Compared to the baseline “low assortment”, both medium assortment (0.568; *p* < 0.001) and high assortment (0.504; *p* < 0.001) show strong, positive, and highly significant effects. This indicates that consumers favour an offer like that of a supermarket or hypermarket, where product variety is ensured. Interestingly, the preference for a medium assortment slightly exceeds that for a high one, suggesting that consumers value variety but not excessive abundance; what matters most is avoiding a perception of scarcity or inconvenience typical of small shops.

The percentage of organic products certified (both more than 50% and equal to 100%) or with a participatory guaranteed scheme of the total products, are positive and statistically significant, showing that consumers assign greater utility to FV configurations with higher proportions of organic or participatory guarantee products. The increase from more than 50% (0.182; *p* = 0.013) to 100% (0.239; *p* = 0.001) confirms a monotonic trend: the higher the share of organic/participatory products, the greater the preference. Thus, ethical and environmental certification represents a key factor of attractiveness.

The coefficient % of local products of the total products (0.352; *p* < 0.001) is also positive and strongly significant. This means that offering a product mix with more than 50% local goods (within 50 km of the FV) significantly increases consumer utility. Local origin, therefore, emerges as one of the main drivers of preference, reflecting the growing importance of territorial proximity and traceability in food choice.

Compared to the reference level (no involvement), all levels of participation are positively valued, although with different levels of significance: medium-low involvement (0.258; *p* = 0.002) is positive and significant; medium-high involvement (0.151; *p* = 0.073) is positive but only marginally significant; high involvement (0.049; *p* = 0.575) is positive but not significant. These results indicate that consumers appreciate participation, but only up to a certain level of involvement. The medium–low participation level is clearly preferred. Conversely, higher levels of involvement, which require regular work inside the FV (three or more hours per month), do not significantly increase utility and, for some segments, even reduce it. It means that consumers are attracted to participatory and relational experiences but tend to resist those that imply continuous or burdensome commitments.

The negative and highly significant coefficient for the “no-choice” constant (asc) is negative and significant (–1.582; *p* < 0.001), indicating that, on average, respondents prefer one of the FV configurations to the “no-choice” option: participants prefer to choose “something” rather than “nothing”. This confirms a general openness and positive attitude toward the FV concept as a whole.

Several attributes show statistically significant standard deviations, confirming that consumer preferences for the FV model are not homogeneous. In particular, the parameters associated with the number of opening days (*p* = 0.007), the proportion of local products (more than 50%, *p* < 0.001), and organic or participatory guarantee products (100%, *p* = 0.020) exhibit strong and significant variability. This suggests that, while the average effects of these attributes are positive, the intensity of preference differs substantially across individuals: some respondents strongly value these features, while others are relatively indifferent.

The significant standard deviation for “no choice” (*p* < 0.001) indicates that the overall propensity to choose one of the proposed FV configurations over the “no-choice” option also varies considerably among consumers. This result reinforces the existence of latent attitudinal factors, possibly linked to different levels of familiarity with alternative food networks or varying degrees of trust in cooperative systems.

Interestingly, the significant heterogeneity detected for the attributes related to consumer involvement (medium-low, *p* = 0.016; medium-high, *p* = 0.039) confirms that participation is a particularly divisive aspect of the FV model. While some respondents perceive participation as an opportunity for engagement and shared responsibility, others consider it a constraint or an additional burden. The same applies to product assortment (high, *p* = 0.050), where preferences diverge between those who appreciate a wide range of products and those who prefer a simpler, more essential offer.

To explore how individual value orientations influence preferences for the FV attributes, we created interaction terms between the three value-based components identified through the PCA and each FV attribute included in the RPL model. These interactions allowed us to examine whether consumers with different value profiles express distinct patterns of preference for the various organizational, ethical, and participatory dimensions of the FV model.

1. Conservation/Self-transcendence (reflecting values of order, stability, relational trust, and collective well-being)—Component 1 (c1).

For respondents belonging to this value profile, preferences are generally consistent with the average trends observed in the model. These individuals value accessibility (more opening days) and local and organic products, which align with their orientation toward social harmony and collective welfare. The significance of heterogeneity terms for several attributes (e.g., opening days per week, medium assortment) suggests that within this group, there is variation related to how traditionalism and relational trust shape purchasing preferences. However, overall, this profile appears as the most aligned with the FV’s cooperative and community-oriented vision.

2. Self-enhancement (expressing values linked to personal success, pleasure, and social status)—Component 2 (c2).

Consumers characterized by self-enhancement values show a lower inclination to engage in participatory activities. The positive and significant coefficient of “no-choice” (0.516; *p* = 0.038) indicates that they are more likely to select the “no-choice” option, signalling weaker interest in joining collective initiatives such as the FV. Moreover, the negative interaction “high involvement” (–0.184; *p* = 0.052) confirms that high levels of involvement (requiring several hours of work or active contribution) reduce their utility. This profile, oriented toward individual gratification and personal achievement, is less attracted by models that emphasize cooperation, reciprocity, or shared governance.

3. Openness to change/Self-transcendence (associated with autonomy, curiosity, innovation, and universalism)—Component 3 (c3).

For individuals characterized by values of openness, autonomy, and universalism, several interactions are negative and significant, specifically: % of organic products more than 50% (–0.202; *p* = 0.010) and equal to 100% (–0.166; *p* = 0.052), medium-high involvement (–0.302; *p* = 0.001), and High involvement (–0.249; *p* = 0.009). These results indicate that, although this segment shares ethical and ecological concerns, it attributes less additional utility to increases in the share of organic/participatory products and to intensive forms of participation. This may be because respondents with such values already assume ecological and ethical coherence as a baseline expectation, while preferring forms of engagement that allow autonomy and flexibility. In this sense, excessive organizational commitment could generate “participation fatigue” among this group. The lower propensity toward organic products in individuals with high Self-transcendence and Openness to change might be explained by a high sensitivity to greenwashing and the elitist perception of organic labels, which conflicts with the egalitarian goals of Universalism. Furthermore, the constant variety-seeking behaviour typical of Openness to change might lead these consumers to prioritize novel food technologies over traditional organic options.

The interactions between value profiles and FV attributes confirm that value orientations significantly condition consumer preferences. The Conservation/Self-transcendence profile appears the most consistent with the cooperative spirit of the FV, while Self-enhancement and Openness/Self-transcendence consumers are more selective: they support the ethical foundations of the model but tend to resist heavy participatory commitments. These insights reinforce the importance of designing flexible and inclusive participation mechanisms to accommodate different motivational structures and enhance the potential scalability of the FV model.

Overall, these interpretations should be read as analytically informed explanations consistent with the observed interaction effects, rather than as direct evidence of underlying psychological or behavioural mechanisms.

The significant standard deviations observed across multiple attributes demonstrate that even within the same value profiles, latent heterogeneity remains relevant, reflecting different degrees of openness, experience, or perceived compatibility with the FV model. Accounting for such unobserved variability strengthens the robustness of the RPL estimates and underlines the need for flexible implementation strategies that can adapt to diverse consumer expectations and engagement capacities.

## 4. Discussion

The results demonstrate that consumers appreciate the ethical and territorial coherence of the FV, its local sourcing, ecological standards, and cooperative governance, provided these features do not compromise convenience and accessibility. The strong preference for moderate participation suggests that individuals value relational and deliberative engagement but reject forms of commitment perceived as excessive or burdensome. This highlights a crucial design principle: participation must be enabling rather than constraining, allowing members to experience belonging and co-responsibility without overloading their daily routines. The observed influence of human values further supports the idea that ethical alignment and value congruence are essential drivers for joining and sustaining community-based food networks. In this sense, the FV can be seen as a hybrid institution, bridging market and community logics, capable of translating ecological and civil economy principles into a practical, scalable food system.

Consistent with previous research on food consumption [27,29,32], the findings reveal that both product-related and organizational attributes significantly influence utility formation. However, beyond functional considerations, the analysis highlights the crucial role of human values in shaping preferences and willingness to participate in sustainability-oriented food systems.

The positive and highly significant coefficients associated with local and organic products demonstrate that environmental and ethical features remain key determinants of consumer choice, in line with earlier evidence on the importance of credence attributes [30,34]. Respondents strongly prefer configurations offering a high proportion of local (within 50 km) and organic or PGS products, supporting the view that trust, transparency, and territorial embeddedness are core drivers of sustainable consumption [13,15]. These findings also align with the principles of the Ecological and Civil Economies, which stress the need to reconnect production and consumption through proximity, reciprocity, and environmental care [8,100].

The results regarding assortment and opening days further confirm the relevance of convenience and accessibility in consumers’ evaluation of alternative food networks. While AFNs are often perceived as limited in terms of product variety and purchasing opportunities [17], respondents in this study expressed a clear preference for configurations that mitigate these constraints. This indicates that, for such models to “jump the scale” [19], they must combine ethical and social innovations with operational efficiency and service quality comparable to conventional retail systems.

The most distinctive aspect of the FV model concerns consumer participation. The results show that respondents value participatory involvement, but only to a moderate extent. Medium–low participation, represented by co-planning of production with farmers, was positively and significantly evaluated, whereas higher levels of engagement requiring regular work inside the FV reduced utility. These finding echoes those of Fitzsimmons and Cicia [35], who observed that ethical commitment does not necessarily translate into a willingness to assume ongoing or labour-intensive responsibilities. Consumers appear to appreciate participation as a symbolic and relational experience [50,51], but excessive involvement may generate what could be termed “participation fatigue,” especially among individuals for whom time is a scarce resource.

The integration of Schwartz’s Theory of Basic Human Values (1992) [41] into the RPL model adds a deeper behavioural interpretation to these results. The significant interactions between value-based components and FV attributes demonstrate that individual motivations are key to understanding heterogeneity in preferences. The Conservation/Self-transcendence profile, oriented toward stability, trust, and collective well-being, shows the strongest alignment with the FV’s cooperative and community-based ethos. These individuals embody the social capital that sustains relational trust and participation, consistent with the moral and relational dimensions emphasized in the CE [8].

Conversely, the Self-enhancement profile, focused on personal success, pleasure, and social status, is associated with a lower likelihood of participation and a higher probability of choosing the “no-choice” option. This group reflects more individualistic orientations, as observed in previous studies linking self-enhancement values with reduced pro-social and pro-environmental behaviours [28,44]. Finally, consumers characterized by Openness to change/Self-transcendence, those valuing autonomy, curiosity, and universalism, display ambivalent preferences: while they support ethical and ecological attributes, they show limited willingness to engage in highly structured or time-consuming participation. This result suggests that idealistic consumers may already internalize ethical coherence as a baseline expectation, preferring flexible and non-hierarchical forms of engagement [48].

Overall, these findings reinforce the conceptual link between Max-Neef’s H-SD (1991) [20] and Schwartz’s value framework. Whereas Max-Neef identifies universal human needs, Schwartz’s theory explains how individuals prioritize and pursue them according to their value orientations. In the context of the FV, participation and ethical consumption emerge as expressions of both axiological needs (affection, participation, identity) and value-driven motivations (self-transcendence, conservation). However, heterogeneity across value profiles indicates that no single participatory model can satisfy all consumers equally.

The findings of this study suggest that the FV model should not be interpreted as a standalone organisational innovation, but rather as a policy-enabled infrastructure embedded within broader Local Food Policies (LFPs). From this perspective, participatory food networks are most effective when supported by place-based governance frameworks that can coordinate multiple actors, integrate sectoral policies, and accommodate heterogeneous value orientations among citizens.

The observed preference for moderate and flexible forms of participation highlights the need for participatory architectures that are enabling rather than constraining. LFPs can address this challenge by designing modular participation schemes that allow citizens to engage at different levels of intensity, ranging from deliberative involvement and co-planning activities to more active roles in production or governance, according to their values, motivations, and time availability. Such flexibility is crucial to avoid participation fatigue while preserving collective responsibility and social embeddedness.

From a governance perspective, these findings support the institutionalisation of local food governance arenas, such as food councils, community food hubs, or cooperative platforms, where public authorities, producers, consumers, civil society organisations, and researchers can jointly define priorities, rules, and evaluation criteria for local food systems. These arenas enable the translation of ethical and ecological values into concrete organisational arrangements, fostering trust, transparency, and long-term commitment.

Policy instruments aligned with this approach include targeted support for community cooperatives, participatory guarantee systems (PGS), and local micro-processing and logistics infrastructures, which reduce entry barriers for small-scale producers while enhancing accessibility and convenience for consumers. Embedding such instruments within regional food strategies and rural–urban development policies can strengthen territorial cohesion, local employment, and the resilience of food systems. More broadly, the results indicate that Local Food Policies should move beyond a uniform conception of citizen participation and instead recognise value-based heterogeneity as a structural condition of contemporary food systems. By combining flexible participation pathways, institutionalised spaces of democratic governance, and integrated policy tools, Local Food Policies can operationalise the principles of the Civil Economy and the food-as-commons approach, supporting the scaling-up of community-based food systems without diluting their ethical and territorial foundations. Table 9 summarises how the main empirical findings of this study can be translated into specific LFP instruments and governance arrangements, highlighting the actors involved in their implementation.

## 5. Conclusions

This study contributes to expanding knowledge on AFNs by integrating the analysis of consumer preferences with individual human values within an innovative and systemic model: the FV. By combining DCE with value-based segmentation, the research provides new insights into how ethical orientations and participation preferences interact in shaping consumer behaviour toward community-based food systems. The findings reveal that consumers seek a balance between ecological coherence, convenience, and meaningful, yet manageable, participation. This multidimensional understanding supports the development of inclusive and resilient food networks rooted in the principles of the Civil and Common Good economies, offering practical pathways for scaling up sustainable food governance models beyond their current niche.

Beyond its empirical contributions, the study advances the debate on transformative food-system strategies by showing how value-based segmentation can serve as a key analytical tool to understand the conditions under which community-driven models can scale without losing their ethical and territorial foundations. By demonstrating that different value orientations correspond to distinct preferences for participation and governance, the findings suggest that scalability does not depend on standardisation, but rather on the capacity of policy-enabled food system models, such as the FV embedded within Local Food Policies, to accommodate diversity through flexible and differentiated engagement pathways.

Despite its contributions, this study has some limitations that should be acknowledged. The analysis was based on stated preferences obtained through a DCE, which, although widely used, may not fully capture actual purchasing behaviour in real market contexts.

At the same time, it is important to acknowledge the inherent complexity of the investigation itself. Although the research design was carefully structured and validated through a pilot phase, some respondents may have found it difficult to fully understand the innovative and multidimensional nature of the FV model, which combines economic, social, and ethical dimensions in a single framework. This may have influenced the way certain attributes, especially those related to consumer involvement, were perceived and evaluated.

Given the non-probability sampling design, the findings are not statistically representative of the Italian population and should not be interpreted in inferential terms. Rather, their external validity lies in the identification of preference patterns and value-based heterogeneity that can inform the design of participatory food system models and guide future empirical research.

To further validate and deepen these findings, a second phase of research is currently being conducted among consumer members of an Italian Food Coop like the FCPS model, whose organizational structures largely reflect the principles of the FV model. While the present study explored consumer preferences in a necessarily hypothetical setting, the follow-up research aims to test whether and how these preferences change when the FV principles are experienced in a real purchasing context. In particular, it is hypothesised that Food Coop members, often already involved as member-workers, will assign greater value to participatory and high-involvement attributes, which were less preferred in the general consumer panel. This comparison will allow us to assess the role of direct engagement and lived experience in shaping the relationship between human values, participation preferences, and support for community-based food systems, thereby strengthening the robustness and external validity of the overall analysis.

## Figures and Tables

**Table 1:** Overview of the main steps of the research process, from case study selection to data analysis.

Steps of the Research Process:	
Case Study Selection	Identification of the FV Model
Discrete Choice Experiment	Definition of attributes and levels of provision
	Experimental design:
	- preliminary experimental design (orthogonal design)
	- testing of the preliminary experimental design
	- priors’ estimation
	- final experimental design (D-efficient design)
	Questionnaire development, including the Schwartz PVQ
	Sampling strategy
Survey	Implementation of the questionnaire survey
Analysis	Reliability analysis of the Schwartz PVQ
	PCA
	RPL model estimation

**Table 2:** Attributes and levels used in the DCE design.

Attributes	Acronym in Model	Levels
Opening days per week	Days	1 day, 3, 5, or 7 days
Product range	Range	Low, Medium, or High
Percentage of organic products certified or with a participatory guaranteed scheme of the total products	%Organic	Less than 50%, more than 50% or 100%
Percentage of local products of the total products	%Local	Less than 50% or more than 50%
Involvement of the consumer partner	Involv	None, Medium-low, Medium-high, or High

**Table 3:** Levels of participation, involvement, and discount.

Levels of Participation	Involvement	Discount
none	none	0
medium-low (participation in co-planning activities)	1 h of work per month + advance payment of 100% of the production costs of the raw materials	10% on co-planned shopping
medium-high (participation in in-store work)	3 h of work per month	20% on standard shopping
high (participation in co-planning activities and in in-store work)	4 h of work per month in total + advance payment of 100% of the production costs of the raw materials	30% on co-planned shopping, and a 20% on standard shopping.

**Table 4:** Example of a choice task.

	Place of Purchase Consistent with the FV Model			
	Option 1	Option 2	Option 3	No Choice
Number of opening days per week	5 days	7 days	1 day	No choice
Product range	High	Low	Medium	
% of organic products	More than 50%	100%	Less than 50%	
% of local products	Less than 50%	More than 50%	Less than 50%	
Consumer partner involvement	None	Medium-high	High	
**Mark an X on the chosen option**				

**Table 5:** Sample statistics (402 individuals).

Consumer Characteristics		Percentage
Gender	Female	50%
	Male	50%
Age	<40	41%
	>40	59%
Occupation	Employed	68%
	Student, housewife, retired	32%
Living area	Urban	85.4%
	Rural	14.6%
Educational level	Primary and Secondary	63.2%
	Higher and University	36.8%
Family income	Low- Medium low	68.6%
	Medium high-High	31.4%

**Table 6:** Schwartz values and Cronbach’s alpha coefficients.

Values	Cronbach’s Alpha
Power	0.624
Achievement	0.758
Benevolence	0.522
Conformity	0.071
Hedonism	0.779
Security	0.545
Self-direction	0.518
Stimulation	0.586
Tradition	0.012
Universalism	0.709

**Table 7:** Principal Component Analysis results.

	Meta-Values		
Values	Component 1	Component 2	Component 3
	Conservation/Self-Transcendence	Self-Enhancement	Openness to Change/Self-Transcendence
Security	**0.808**	0.085	0.113
Conformity	**0.755**	−0.019	−0.005
Benevolence	**0.682**	0.097	0.345
Tradition	**0.654**	0.102	0.230
Power	−0.014	**0.851**	−0.195
Achievement	0.147	**0.817**	0.150
Hedonism	0.075	**0.696**	0.388
Self-direction	0.338	0.076	**0.767**
Stimulation	−0.034	0.492	**0.734**
Universalism	0.507	−0.223	**0.669**

Variance explained: 69.86%.

**Table 8:** DCE model results considering the PCA.

Coefficient	Coefficients’ Estimate			Standard Deviation of Random Parameters Distribution ^§^		
	Estimate	Std.Err.		Estimate	Std.Err.	
**Days**	**0.1357514**	**0.0165789**	***	0.1113336	0.0413909	**
Days#c1	−0.0306834	0.0201288		0.1331439	0.034749	***
Days#c2	−0.0104131	0.0171014		−0.0669785	0.0638717	
Days#c3	−0.0302141	0.0185066		−0.0811502	0.0341544	*
**Range_medium**	**0.568428**	**0.0745082**	***	−0.0056293	0.2180672	
Range_medium#c1	0.0128305	0.0791665		0.2498558	0.1271636	*
Range_medium#c2	0.0852183	0.0743751		0.0218622	0.2261908	
Range_medium#c3	−0.0699386	0.0778913		0.0399143	0.1925089	
**Range_high**	**0.5041672**	**0.0778941**	***	0.3271325	0.1669547	.
**Range_high#c1**	**−0.1475861**	**0.0798633**	**.**	−0.0815044	0.197527	
Range_high#c2	0.0608038	0.077776		0.1193572	0.3064297	
**Range_high#c3**	**−0.149047**	**0.0802059**	**.**	−0.0118289	0.1977269	
**%Organic_>50**	**0.182432**	**0.0732625**	*	−0.3441646	0.1682607	*
%Organic_>50#c1	0.0562445	0.0814898		0.2850279	0.16404	.
%Organic_>50#c2	0.0160626	0.0755704		0.1254942	0.1729656	
**%Organic_>50#c3**	**−0.201983**	**0.0784306**	*	0.1938627	0.1688932	
**%Organic_100**	**0.2389888**	**0.0745948**	**	0.4110702	0.1768477	*
%Organic_100#c1	−0.0055412	0.0790969		0.215484	0.1695997	
%Organic_100#c2	−0.0009329	0.0763247		−0.0408786	0.158359	
**%Organic_100#c3**	**−0.1658139**	**0.0852949**	**.**	0.4623954	0.1531734	**
**%Local_high**	**0.3519379**	**0.0680552**	***	0.5555975	0.1452155	***
%Local_high#c1	−0.0207961	0.0714659		0.0127376	0.1610745	
%Local_high#c2	0.024354	0.0703522		−0.1782152	0.2719463	
%Local_high#c3	−0.0337837	0.0766988		0.4307464	0.15916	**
**Involv_low**	**0.2577138**	**0.0848941**	**	−0.3576972	0.1481461	*
Involv_low#c1	−0.027055	0.0937946		0.4353753	0.1540397	**
Involv_low#c2	0.1271551	0.0882214		0.1389385	0.1647598	
Involv_low#c3	−0.0655865	0.0905269		0.0504118	0.1620968	
**Involv_high**	**0.1513179**	**0.0843424**	**.**	−0.4347242	0.2099674	*
Involv_high#c1	0.0227018	0.0904619		0.1908358	0.213956	
Involv_high#c2	−0.0885897	0.0869441		0.1639399	0.3971026	
**Involv_high#c3**	**−0.3015378**	**0.0913035**	***	0.0773517	0.2137314	
Involv_elev	0.0494753	0.088299		0.3875361	0.2542993	
Involv_elev#c1	0.0090868	0.0932565		−0.2957081	0.1958496	
**Involv_elev#c2**	**−0.1839165**	**0.0946316**	**.**	0.462663	0.1572633	**
**Involv_elev#c3**	**−0.2479488**	**0.0947618**	**	−0.0823791	0.2762441	
**asc**	**−1.5821296**	**0.3267352**	***	2.6143741	0.3020607	***
asc#c1	−0.0170639	0.2085303		0.6336262	0.2355964	**
**asc#c2**	**0.5163716**	**0.2488423**	*	−0.0989615	0.453152	
**asc#c3**	**−0.388464**	**0.2199106**	**.**	0.8705771	0.2447473	***
Observations:	9,648					
Subjects:	402					
Log-Likelihood:	−2804.178					
McFadden pseudo-R^2^	0.161					
AIC:	5768.356					
BIC	6231.413					

Significance: 0 ‘***’ 0.001 ‘**’ 0.01 ‘*’ 0.05 ‘.’ 0.1 ‘ ‘ 1. ^§^ Random parameters were assumed as normally distributed. Negative standard deviations should be interpreted as positive [99]. The interacted variables are indicated with the “#” symbol. c1, c2 and c3 represent Component 1, Component 2 and Component 3 of Table 7.

**Table 9:** Mapping empirical results to Local Food Policy instruments.

Empirical Results from the Study	Local Food Policy Instruments	Actors Involved
Strong preference for local and organic products; importance of trust and territorial embeddedness	Support for short supply chains; participatory certification schemes (e.g., PGS); local procurement policies	Local authorities; farmers; consumer groups; certification bodies; civil society
Preference for moderate participation; rejection of labour-intensive commitments	Modular participation schemes; differentiated membership models; co-planning platforms	Consumers; cooperatives; food hubs; local administrations
Risk of participation fatigue when engagement is perceived as burdensome	Institutionalised food governance spaces (food councils, community food platforms) enabling deliberation without compulsory labour	Municipalities; regions; civil society organisations; research institutions
Importance of convenience, assortment, and accessibility	Investments in local logistics, micro-processing, and distribution infrastructures	Local governments; cooperatives; producer organisations; SMEs
Value-based heterogeneity among consumers (Schwartz profiles)	Inclusive governance designs recognising diverse motivations and capacities	Public authorities; community organisations; consumer representatives
Alignment between self-transcendence values and cooperative participation	Education and training programmes on food citizenship and cooperative entrepreneurship	Schools; universities; NGOs; cooperatives; local authorities
Need to “jump the scale” without losing ethical coherence	Integration of FV-like initiatives into regional food strategies and rural–urban development policies	Regions; municipalities; policymakers; multi-actor partnerships

## Data Availability

The original contributions presented in this study are included in the article. Further inquiries can be directed to the corresponding author.

## Abbreviations

The following abbreviations are used in this manuscript:

CE	Civil Economy
H-SD	Human-Scale Development
ECG	Economy for the Common Good
AFNs	Alternative Food Networks
CSA	Community Supported Agriculture
SPGs	Italian Solidarity Purchase Groups
OGSD	Organized Groups of Supply and Demand
FCPS	Food Coop Park Slope
FV	Food Village
DCE	Discrete Choice Experiment
PVQ	Schwartz Portrait Values Questionnaire
SVS	Schwartz Value Survey
WTP	willingness to pay
SHV	Schwartz Human Value
RPL	Random Parameters Logit
PCA	Principal Component Analysis
PGS	Participatory Guarantee System

## Appendix A

**Table A1:** Schwartz values conceptual definition and goals.

Basic Values	Conceptual Definition	Values
Self-direction	Independent thought and action-choosing, creating, exploring	creativity, freedom, independence, curiosity, choosing own goals, self-respect, intelligent, privacy
Stimulation	Excitement, novelty, and challenge in life	a varied life, an exciting life, daring
Hedonism	Pleasure and sensuous gratification for oneself	pleasure, enjoying life, self-indulgent
Achievement	Personal success through demonstrating competence according to social standards	success, capable, ability, ambitious, influential, intelligent, self-respect, social recognition
Power	Social status and prestige, control or dominance over people and resources	social power, authority, wealth, preserving my public image, social recognition
Security	Safety, harmony, and stability of society and of relationships	family security, national security, social order, clean, reciprocation of favours, healthy, moderate, sense of belonging
Conformity	Restraint of actions, inclinations, and impulses likely to upset or harm others and violate social expectations or norms	obedient, self-discipline, politeness, honouring parents and elders, loyal, responsible
Tradition	Respect, commitment, and acceptance of the customs and ideas that one’s culture or religion provides	respect for tradition, humble, devout, accepting one’s portion in life, humility, moderate, spiritual life
Benevolence	Preservation and enhancement of the welfare of people with whom one is in frequent personal contact	helpfulness, honesty, forgivingness, loyalty, true friendship, mature love, sense of belonging, meaning in life, a spiritual life
Universalism	Understanding, appreciation, tolerance, and protection for the welfare of all people and for nature	broadmindedness, social justice, equality, world at peace, world of beauty, unity with nature, wisdom, protecting the environment, inner harmony, a spiritual life

From Knoppen e Saris (2009) [57] with slight modification.

**Table A2:** Schwartz’s 21-item PVQ.

Description of People	To Which Extent This Person Is like You?					
	Very Much like Me	Like Me	Some-what like Me	A Little like Me	Not like Me	Not Like Me At All
Thinking up new ideas and being creative is important to him/her. He/she likes to do things in his own original way.	1	2	3	4	5	6
It is important to him/her to be rich. He/she wants to have a lot of money and expensive things.	1	2	3	4	5	6
He/she thinks it is important that every person in the world should be treated equally. He/she believes everyone should have equal opportunities in life.	1	2	3	4	5	6
It is important to him show his/her abilities. He/she wants people to admire what he/she does.	1	2	3	4	5	6
It is important to him/her to live in secure surroundings. He avoids anything that might endanger his/her safety.	1	2	3	4	5	6
He/she likes surprises and is always looking for new things to do. He/she thinks it is important to do lots of different things in life.	1	2	3	4	5	6
He/she believes that people should do what they’re told. He/she thinks people should always follow rules, even when no-one is watching	1	2	3	4	5	6
It is important to him/her to listen to people who are different from him/her. Even when he/she disagrees with them, he/she still wants to understand them.	1	2	3	4	5	6
It is important to him/her to be humble and modest. He/she tries not to draw attention to him/herself.	1	2	3	4	5	6
Having a good time is important to him/her. He/she likes to “spoil” him/herself.	1	2	3	4	5	6
It is important to him/her to make his/her own decision about what he/she does. He/she likes to be free and not depend on others.	1	2	3	4	5	6
It is very important to him/her to help the people around him/her. He/she wants to care for their well-being	1	2	3	4	5	6
Being very successful is important to him/her. He/ she hopes people will recognise his/her achievements.	1	2	3	4	5	6
It is important to him/her that the government ensures his/her safety against all threats. He/she wants the state to be strong so he/she can defend its citizens.	1	2	3	4	5	6
He/she looks for adventures and likes to take risks. He/she wants to have an exciting life.	1	2	3	4	5	6
It is important to him/her always to behave properly. He/she wants to avoid doing anything people would say is wrong	1	2	3	4	5	6
It is important to him/her to get respect from others. He/she wants people to do what he/she says.	1	2	3	4	5	6
It is important to him/her to be loyal to his/her friends. He/she wants to devote him/herself to people close to him/her.	1	2	3	4	5	6
He/she strongly believes that people should care for nature. Looking after the environment is important to him/her	1	2	3	4	5	6
Tradition is important to him/her. He/she tries to follow the customs handed down by his religion or his/her family	1	2	3	4	5	6
He/she seeks every chance he/she can to have fun. It is important to him/her to do things that give him/her pleasure.	1	2	3	4	5	6

Capanna et al., (2005) [55]; Caprara et al., (2011) [56].
